# A Molecular Modeling Investigation of the Therapeutic Potential of Marine Compounds as DPP-4 Inhibitors

**DOI:** 10.3390/md20120777

**Published:** 2022-12-13

**Authors:** Priya Antony, Bincy Baby, Hamda Mohammed Aleissaee, Ranjit Vijayan

**Affiliations:** 1 Department of Biology, College of Science, United Arab Emirates University, Al Ain P.O. Box 15551, United Arab Emirates; 2 Department of Chemistry, College of Science, United Arab Emirates University, Al Ain P.O. Box 15551, United Arab Emirates; 3 The Big Data Analytics Center, United Arab Emirates University, Al Ain P.O. Box 15551, United Arab Emirates; 4 Zayed Center for Health Sciences, United Arab Emirates University, Al Ain P.O. Box 17666, United Arab Emirates

**Keywords:** diabetes, DPP-4, marine compounds, molecular docking, molecular dynamics

## Abstract

Type 2 diabetes mellitus (T2DM) is a chronic metabolic disorder characterized by elevated levels of blood glucose due to insulin resistance or insulin-secretion defects. The development of diabetes is mainly attributed to the interaction of several complex pathogenic, genetic, environmental and metabolic processes. Dipeptidyl peptidase-4 (DPP-4) is a serine protease that cleaves X-proline dipeptides from the N-terminus of several polypeptides, including natural hypoglycemic incretin hormones. Inhibition of this enzyme restores and maintains glucose homeostasis, making it an attractive drug target for the management of T2DM. Natural products are important sources of bioactive agents for anti-T2DM drug discovery. Marine ecosystems are a rich source of bioactive products and have inspired the development of drugs for various human disorders, including diabetes. Here, structure-based virtual screening and molecular docking were performed to identify antidiabetic compounds from the Comprehensive Marine Natural Products Database (CMNPD). The binding characteristics of two shortlisted compounds, CMNPD13046 and CMNPD17868, were assessed using molecular dynamics simulations. Thus, this study provides insights into the potential antidiabetic activity and the underlying molecular mechanism of two compounds of marine origin. These compounds could be investigated further for the development of potent DPP-4 inhibitors.

## 1. Introduction

Type 2 Diabetes Mellitus (T2DM) is a disorder characterized by defective insulin secretion or insulin resistance [1]. Alarmingly, its global prevalence is reaching epidemic proportions. Based on the International Diabetic Federation Report (IDF), diabetes is classified as one of the fastest-growing emergencies, with more than half a million people living with this condition. Recent IDF projections indicate that the number is expected to reach around 643 million by 2030 and 783 million by 2045 (IDF 2021). Dysregulation of glucose homeostasis is the key factor and is influenced by several pathophysiologic defects, including insulin resistance, pancreatic cell dysfunction and excessive hepatic glucose production [2]. Long-term diabetic conditions will eventually lead to significant diabetic complications, including neuropathy, nephropathy, cardiovascular diseases (CVD) and retinopathy [3].

Drug therapy for treating T2DM has progressed significantly in recent years. At present, dipeptidyl peptidase-4 (DPP-4) inhibitors are widely used in initial monotherapy or combination therapy for treating diabetes [4]. DPP-4 is a multifunctional glycoprotein possessing N-terminal serine dipeptidase activity and is found ubiquitously on the cell surface. This protein plays a key role in the clearance of a variety of bioactive peptides including incretin hormones, glucagon-like peptide 1 (GLP-1) and glucose-dependent insulinotropic peptide (GIP). Both intestinal peptide hormones maintain glucose homeostasis by inducing insulin secretion and suppressing glucagon production [5]. Due to the ability of DPP-4 to cleave the GLP-1 hormone, inhibitors that potentiate the effects of the incretin hormones were explored for the treatment and management of diabetes [6]. Several DPP-4 inhibitors, including sitagliptin, vildagliptin, alogliptin and saxagliptin, are widely used as oral antidiabetic agents [7]. However, side effects, lifelong dependence and economic burden are limiting factors. Therefore, identifying novel DPP-4 inhibitors from natural sources with lower side effect would add to the arsenal of accessible hypoglycemic drugs.

Marine ecosystems have attracted a great deal of attention due to the rich and wide biodiversity they host [8]. Many marine resources have been explored by researchers to develop novel biologically active scaffolds for the production and development of antidiabetic drugs [9]. Marine natural products have diverse structural characteristics compared to terrestrial natural products, since they are exposed to a wide range of environmental factors that differ from those of terrestrial plants. Harsh marine conditions favor the production of molecules with unique structures in terms of diversity, structure, and functional features. There is also evidence of higher incidence of significant bioactivity when compared to natural products from terrestrial life forms [10,11]. Several clinical studies have also highlighted the potential of the marine environment in drug discovery. For instance, the peptide toxin ω-conotoxin, derived from a marine snail, is used as an analgesic drug under the tradename Prialt [12]. Similarly, ecteinascidin-743 (ET-743), a marine alkaloid derived from tunicate *Ectenascidia turbinate*, is an FDA-approved anticancer drug [13]. The terpene dysidine, found in the Hainan sponge *Dysidea villosa*, effectively activates the insulin signaling pathway by inhibiting protein tyrosine phosphatase 1B (PTP1B) and has successfully entered preclinical trials [14]. Thus, the bioactive potential of marine compounds in the drug discovery and development process is quite significant.

Virtual screening and molecular docking are computational techniques applied to identify lead molecules starting from a huge and diverse library of chemical compounds. Compared to traditional experimental high-throughput screening (HTS), virtual screening is a rational drug-discovery approach that has the advantage of being fast, low cost and effective [15]. Here, compounds from the Comprehensive Marine Natural Products Database (CMNPD) were virtually screened to predict and identify potential antidiabetic compounds that could bind to and inhibit DPP-4 [16]. Further, the stability of the bound complexes was assessed using molecular dynamics (MD) simulation studies.

## 2. Results and Discussion

**Figure 1:**
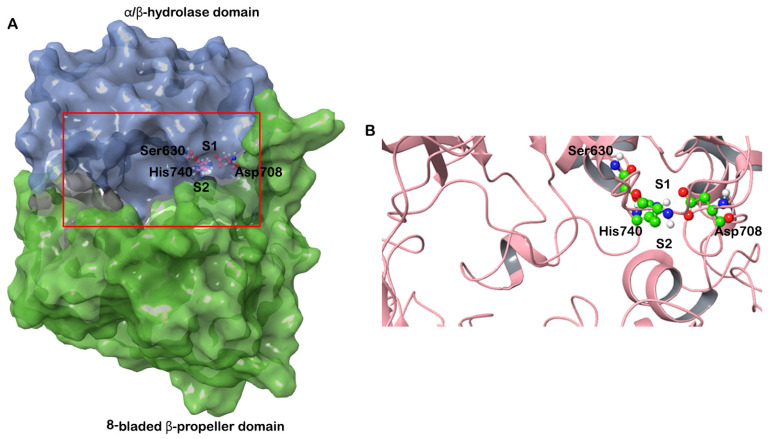
(**A**) Structure of human DPP-4 depicted in surface representation. The β-propeller domain is colored green and the α/β hydrolase domain is colored blue. The S1 and S2 pockets are also marked. The catalytic triad (Ser630, Asp708 and His 740) is shown in ball and stick representation. (**B**) The boxed region in (**A**) is enlarged to show the active site and the catalytic triad.

To confirm the validity of the results, molecular docking was performed with three structures of DPP-4 (PDB IDs: 6B1E, 5I7U, 5T4E). The three structures were crystallized with different ligands bound. The root mean square deviation (RMSD) of the structures 6B1E and 5I7U is 0.13 Å, 6B1E and 5T4E is 0.17 Å and 5I7U and 5T4E is 0.11 Å. The structure of DPP-4 consists of two domains—an N terminal 8-bladed β-propeller domain and a C-terminal α/β hydrolase domain. The binding pocket of DPP-4 involves a catalytic triad consisting of Ser630, Asp708 and His740; an oxyanion cavity containing Tyr47 and Ser631; a hydrophobic S1 pocket consisting of residues Tyr631, Val656, Trp659, Tyr662, Tyr666, and Val711; and a charged S2 pocket consisted of residues Arg125, Glu205, Glu206, Phe357, Ser209 and Arg358 (Figure 1) [17].

**Figure 2:**
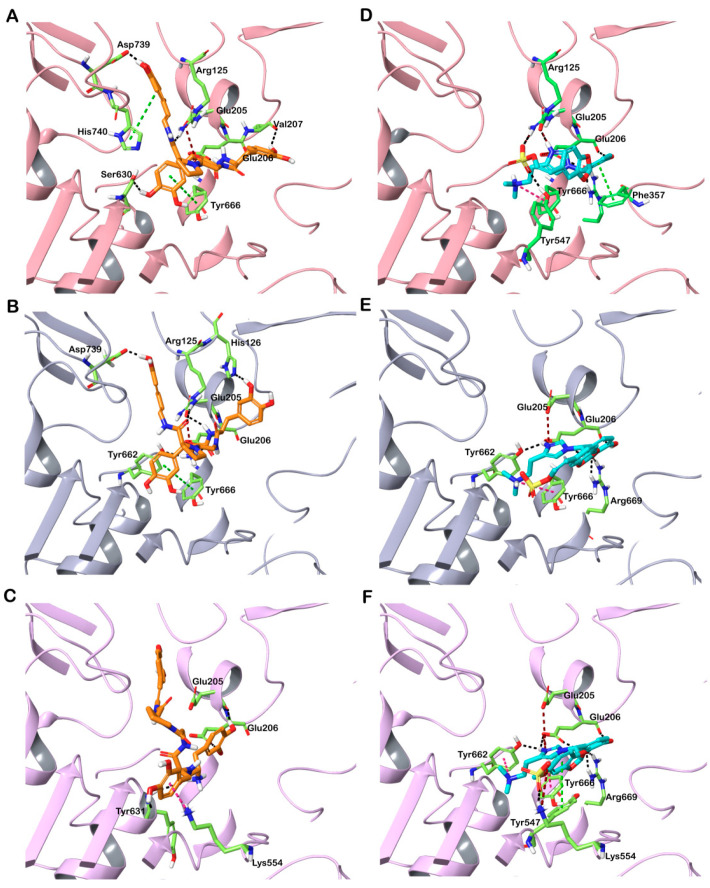
Interaction of docked CMNPD13046 (orange stick representation) and CMNPD17868 (cyan stick representation) with DPP-4. (**A**) 6B1E-CMNPD13046; (**B**) 5I7U-CMNPD13046; (**C**) 5T4E-CMNPD13046; (**D**) 6B1E-CMNPD17868; (**E**) 5I7U-CMNPD17868; and (**F**) 5T4E-CMNPD17868. Hydrogen bonds, π-π interactions, π-cation and salt bridges are represented as black, green, pink and red dashed lines, respectively.

Here, all conformers of the molecules from the CMNPD were flexibly docked into the active site of the DPP-4 structures. Finally, the best interacting compounds were ranked based on GlideScore and the best pose of the ligand was chosen. Out of 31,561 compounds screened, two molecules—CMNPD13046 and CMNPD17868—exhibited good GlideScores and binding free energy calculated using the molecular mechanics-generalized born surface area (MM-GBSA) approach. The top-ranked compounds that docked to DPP-4 are shown in Table 1, along with the drug molecule vildagliptin. CMNPD13046 docked to the three DPP-4 structures in a similar manner (Supplementary Figure S2A). It bound to the active site of the DPP-4 structure 6B1E with a GlideScore of −13.34 kcal/mol and MM-GBSA-based binding energy of −74.92 kcal/mol. This molecule formed hydrogen bonds with Arg125, Glu206, Val207, Ser630 and Asp739 that may play an important role in stabilizing the complex. Apart from these interactions, this molecule formed several interactions with the hydrophobic residues deep in the active site. Additionally, π-π stacking was also observed with the residues Tyr666 and His740. The molecule also interacted with DPP-4 by forming salt bridges with Glu205 and Glu206 from the S2 subsite. The formation of hydrogen bonds and salt bridge with Glu206 residue enhances the binding efficiency of the inhibitor molecule (Figure 2A) [18]. In the structure 5I7U, the compound CMNPD13046 bound to the active site in a pose similar to the docked pose in 6B1E by forming hydrogen bonds, hydrophobic interactions, π-π interactions and salt bridges (Figure 2B). It had a GlideScore of −12.78 kcal/mol and binding energy of −70.77 kcal/mol, respectively. Likewise, in the 5T4E-CMNPD13046 complex, the GlideScore and binding energy were found to be −11.4 kcal/mol and −79.27 kcal/mol. Moreover, the binding pose and interactions of CMNPD13046 were consistent in all three structures, which lends further support to the results (Figure 2C). The bioactive molecule CMNPD13046 is tunichrome Sp-1, a modified pentapeptide found in the hemocytes of the ascidian *Styela plicata*, with the structure H-DOPA-DOPA-Gly-Pro-dcΔDOPA, where DOPA = 3,4-dihydroxyphenylalanine and dcΔDOPA = decarboxy-(E)-α,β-dehydro-DOPA [19,20]. It is a low-molecular-weight linear peptide with a number of properties including metal chelation, sequestration of vanadium or iron, wound healing and antibacterial activity [21,22]. Tunichrome sp-1 interacted in a similar binding pose in all three docked structures, whereby the N-terminal region dcΔDOPA interacted with Glu205 and Glu206 by forming hydrogen bonds and salt bridges. These glutamate residues are highly conserved amino acids across the DPP family and they are essential for its enzymatic activity [23]. The combination of charge–charge and hydrogen bonding electrostatic interactions with these residues allows the molecule to bind effectively to the S2 pocket of the DPP-4 [18]. Apart from this interaction, the N-terminal aromatic ring of the molecule formed π-π stacking with Tyr666 in the S1 pocket. Additionally, the C-terminal carbonyl group of the molecule interacted with the amino group of Arg125 residing in the hydrophobic region. Interactions with these critical residues have been reported to be significant for DPP-4 inhibitory activity [24].

CMNPD17868 docked to the three DPP-4 structures in a similar pose (Supplementary Figure S2B). It bound to the DPP-4 structure 6B1E by interacting with key residues in the active site of the protein. The complex formed with a GlideScore of −13.45 kcal/mol and binding energy of −73.08 kcal/mol. CMNPD17868 formed four hydrogen bonds with Glu205, Glu206, Tyr547 and Arg669 residues. The formation of numerous hydrophobic interactions, salt bridges, π-π and π-cation interactions determine the strong binding of the CMNPD17868 to the protein (Figure 2D). Similar to 6B1E, in the other two structures (517U and 5T4E), the molecule CMNPD17868 bound to the active site of the proteins with GlideScores of −11.68 kcal/mol and −11.51 kcal/mol, respectively (Table 1). In all three structures, molecule CMNPD17868 bound to the active site with a similar orientation and interactions (Figure 2E,F). The hydrophobic interactions are illustrated in Supplementary Figure S1. This marine molecule is an imidazole derivative isolated from an association of the sponges *Poecillatra wondoensis* and *Jaspis* sp. Genus *Jaspis* is widely recognized as a rich source of bioactive molecules including aromatics, peptides, amino-acid derivatives and modified nucleosides [25]. In all three DPP-4 structures, it was observed that the 3,4-dihydroxy phenyl group of this molecule interacted with Glu206 by forming a hydrogen bond and imidazole moiety that interacted with Glu205, Glu206 and Tyr666 by forming hydrogen bonds, salt bridge and π–cation interaction. As described earlier, this network of salt bridges, hydrogen and hydrophobic interactions helps the molecule to stabilize inside the S2 pocket of the DPP-4 active site [18]. It was reported that DPP-4 could accommodate a diverse set of inhibitors, as it possesses a large active site cavity (diameter ≥ 20 Å) along with multiple binding subsites. A comprehensive assessment of the binding mode of DPP-4 inhibitors concluded that binding with residues such as Arg125, Glu205, Glu206, Tyr662 and Asn710 was common among several inhibitors. This indicates that involving these residues could be crucial for stable binding and enzyme inhibition [26]. For a comparative analysis, vildagliptin—a commercially available inhibitor—was docked to the active site of DPP-4. The drug molecule bound the protein by forming hydrogen bonds and salt bridges with the catalytic residues Glu205 and Glu206 found in the S2 site. Furthermore, the drug exhibited a lower GlideScore and binding energy compared to the screened marine molecules (Table 1).

**Figure 3:**
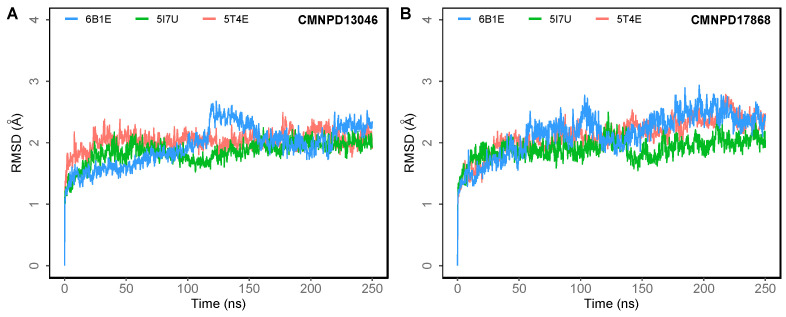
Root mean square deviation (RMSD) of Cα atoms of DPP-4 in 250 ns MD simulations of DPP-4 complexed with (**A**) CMNPD13046; (**B**) CMNPD17868.

**Figure 4:**
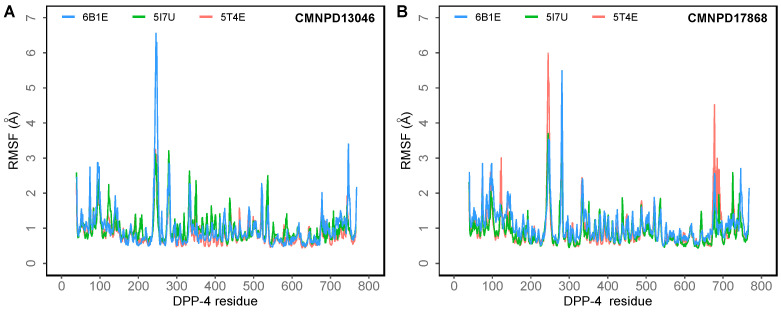
The root mean square fluctuation (RMSF) of the Cα atoms of the DPP-4 in 250 ns MD simulations of the DPP-4 complexed with (**A**) CMNPD13046; (**B**) CMNPD17868.

**Figure 5:**
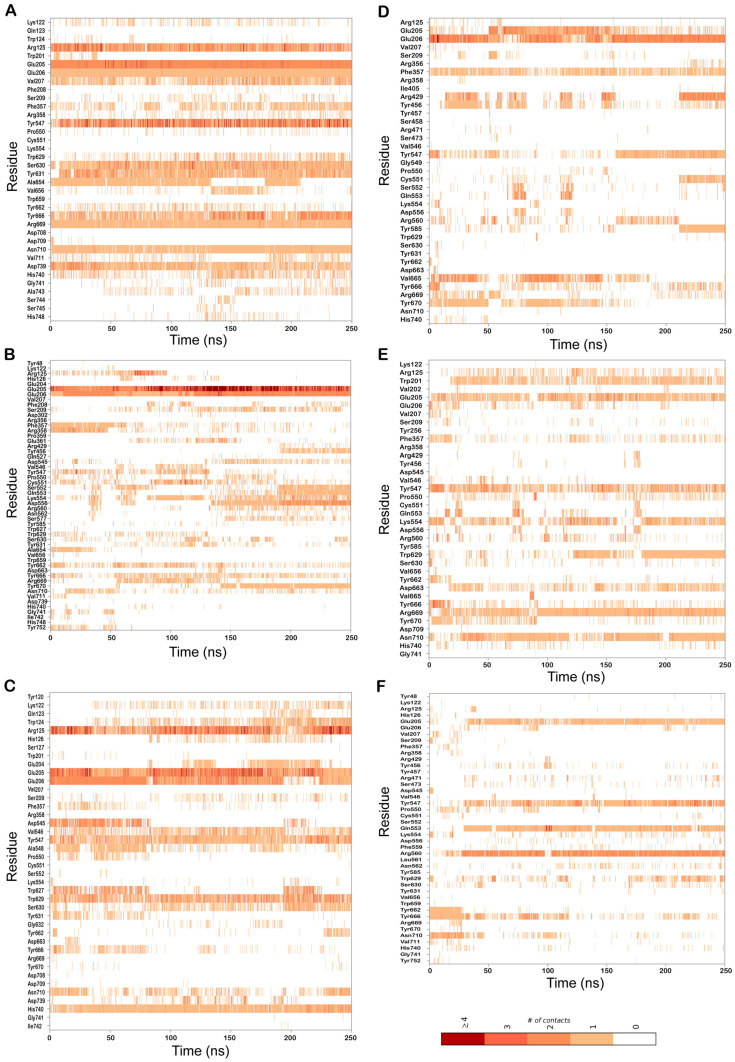
Residues of DPP-4 that interact with the bound ligand over the course of the 250 ns MD simulations in complexes (**A**) 6B1E-CMNPD13046; (**B**) 5I7U-CMNPD13046; (**C**) 5T4E-CMNPD13046; (**D**) 6B1E-CMNPD17868; (**E**) 5I7U-CMNPD17868; (**F**) 5T4E-CMNPD17868. Multiple contacts with the same residue are indicated by darker bars, as indicated by the color bar.

To understand the dynamics of the protein–ligand complex, molecular dynamics (MD) simulations were performed for 250 ns. This technique permits introspection of structural stability, flexibility and conformational behavior of protein–ligand complexes. Here, root mean square deviation (RMSD), root mean square fluctuation (RMSF), radius of gyration (Rg) and various interactions of the protein–ligand complexes were assessed. RMSD of the Cα atoms of the complexes revealed the rigidity and dynamic stability of the complexes. In the DPP-4-CMNPD13046 complexes, after an initial rise, complexes converged and reached a stable RMSD below 3 Å (Figure 3A). The complexes involving the structures 5I7U and 5T4E reached a stable state after 20 ns, whereas the 6B1E exhibited some degree of deviation during the simulation. Nonetheless, all three complexes maintained stable equilibrated structures throughout the simulation period with RMSD below 3 Å (Figure 3B). To determine protein regions exhibiting greater flexibility, the RMSF of Cα atoms were calculated and plotted. While RMSD gives an indication of overall structural deviations, RMSF provides an indication of residue level fluctuations within the protein. In complexes of both compounds (CMNPD13046 and CMNPD17868), higher flexibility was observed in poorly organized regions involving loops, coils and turns, while well-structured α-helices and β-sheets exhibited low RMSF profiles indicating stable secondary structure (Figure 4). The compactness of the complexes was also calculated by determining the radius of the gyration (Rg) profile of the proteins. In complexes involving both ligands, Rg was maintained at a stable value, indicating the retention of a stable compact structure. The protein–ligand interactions including hydrogen bonds, hydrophobic bonds, ionic bonds and water bridges play a significant role in protein–ligand binding and stability. During the course of the 250 ns simulation, it was observed that numerous interactions intermittently formed and broke between DPP-4 and the bound ligand. Interestingly, most of the crucial interactions observed during the docking process were found to be retained throughout the simulation period (Figure 5). During the course of the simulation, ligand CMNPD13046 bound to the active site of DPP-4 by preserving sustained interactions with Arg125, Glu205 and Glu206 residues. Similar to this, in all three protein structures, CMNPD17868 retained the interactions with the critical residues involved in the S2 pocket, such as Glu205 and Glu206. In addition to this, the ligand retained interaction with Tyr547, an important residue involved in catalytic activity [27]. The MM-GBSA-based binding free energy was computed using snapshots taken every 25 ns from the simulation trajectories. For the CMNPD13046 bound complexes, the computed binding free energies were −86.2 ± 9.1 kcal/mol (5I7U), −90.4 ± 9.3 kcal/mol (5T4E), and −105.0 ± 9.6 kcal/mol (6B1E) and for CMNPD17868 bound complexes, the binding free energies were −61.4 ± 6.3 kcal/mol (5I7U), −45.8 ± 6.5 kcal/mol (5I7U), and −46.6 ± 6.1 kcal/mol (6B1E). These strong and stable interactions of the docked ligands in the active site of the protein support their potential to be good DPP-4 inhibitors.

## 3. Materials and Methods

### 3.1. Target Selection and Protein Preparation

Three-dimensional (3D) structures of DPP-4 used for the docking were obtained from the Protein Data Bank (PDB) [28]. The PDB IDs selected for the study were 6B1E, 5I7U and 5T4E. The three structures were co-crystallized with different ligands and the three structures were used to validate the docking results. The protein structures were preprocessed using the Protein Preparation Wizard of the Schrödinger suite of programs [29]. This step was performed to remove crystallographic water molecules, add and optimize hydrogens, simplify multimeric complexes, create disulfide bonds, adjust formal charges and bond orders of atoms that are attached to metal ions and cofactors, fix the orientation of misoriented groups, optimize and, finally, minimize the structures for docking using the OPLS 2005 forcefield [30,31].

### 3.2. Receptor Grid Generation

The receptor grid was generated around the centroid of active site residues of DPP-4 with a van der Waal’s scaling factor of 1.0 and a partial charge cut-off of 0.25. While generating the receptor grid, no constraints were used. The rest of the parameters were set to default. The OPLS force field 2005 was used to model the protein–ligand interactions [32].

### 3.3. Ligand Preparation

The Comprehensive Marine Natural Products Database (CMNPD) (https://www.cmnpd.org, accessed on 20 September 2022) was screened to identify potential ligands of DPP-4. CMNPD contains 31,561 distinct natural marine chemicals from over 13,000 organisms [16]. Schrödinger LigPrep was used to prepare the structures of these ligands for docking. This involved the conversion of ligand 2D structures to 3D, the addition of hydrogen atoms, generation of various ionization states and tautomers and, finally, optimization of the geometries [33]. Vildagliptin, a potent selective inhibitor of DPP-4, was also retrieved from PubChem and used for comparison [34].

### 3.4. Virtual Screening and Free Energy of Binding

Grid-based molecular docking was set up using the Schrödinger software suite to predict the binding orientation and interaction of the ligands. The processed compounds from CMNPD were screened using a virtual screening workflow which includes three stages: (1) high-throughput virtual screening (HTVS), (2) standard precision (SP) and extra-precision (XP) docking [35,36]. The compounds retained from the HTVS stage were passed to the next stage, SP docking; the SP selected compounds were then docked using the more accurate and computationally intensive XP mode. At each stage, the top 10% of compounds were retained and proceeded to the next stage. Finally, the best compounds were selected based on XP GlideScore, a scoring scheme used to report the strength of the binding of a ligand to a protein. Protein–ligand interactions, including hydrogen bonds, hydrophobic interactions, π-π stacking and cation-π interactions, were assessed. The free energies for the binding of ligands with the proteins (ΔG_bind_ in kcal/mol) were calculated by using the MM-GBSA method. For each DPP-4-ligand complex, the MM-GBSA-based binding free energy was estimated using the equation: ΔG_bind_ = G_complex_ − G_protein_ − Gl_igand_, where ΔG_bind_ is the binding free energy and G_complex_, G_protein_, and G_ligand_ are the free energies of complex, protein and ligand, respectively [37].

### 3.5. Molecular Dynamics

To examine the dynamics and stability of the protein–ligand complex, docked poses from the XP docking stage were subjected to 250 ns MD simulations using Desmond employing the OPLS force field. MD permits the assessment of the structural dynamics of the docked complex to evaluate the extent of inter and intra-molecular associations and complex stability. Structures of ligands bound to DPP-4 were placed in orthorhombic boxes 90 Å × 90 Å × 90 Å in size and solvated with single-point charge (SPC) water molecules using the Desmond System Builder (Schrödinger, LLC, New York, NY, USA). The systems were neutralized with counterions and a salt concentration of 0.15 M NaCl was maintained. The OPLS forcefield was used for all calculations and all systems were subjected to Desmond’s default eight-stage relaxation protocol before the start of the production run. For the simulations, the isotropic Martyna–Tobias–Klein barostat and the Nosé–Hoover thermostat were used to maintain the pressure at 1 atm and temperature at 300 K, respectively [38,39]. The short-range cutoff was set as 9.0 Å and long-range coulombic interactions were evaluated using the smooth particle mesh Ewald method (PME) [40]. Finally, simulation trajectories were analyzed to identify the stability of the protein–ligand interactions and the integrity of the complex.

## 4. Conclusions

T2DM has attained the status of a global pandemic and its prevalence is rising dramatically. DPP-4 is a major target for diabetes, as its inhibitors potentiate the effects of the incretin hormones. Due to high structural diversity and bioactivity, marine natural products are promising sources for discovering new leads for the drug-discovery process. From molecular docking studies, the marine molecules CMNPD13046 and CMNPD17868, from the CMNPD database, exhibited a better binding score and binding energy than a commercially available drug molecule. Stable interactions with key residues in the active site were also observed in 250 ns MD simulations of the complexes. These molecules could be further explored in in vitro and in vivo studies. Thus, this study provides early insights into the mechanism by which these marine molecules could have a positive effect on diabetics.

## Figures and Tables

**Table 1:** Docking score, binding energy and interaction of docked marine compounds and vildagliptin with DPP-4.

DPP-4 PDB ID	Docked Molecule	XP GlideScore (kcal/mol)	MM-GBSA Binding Energy (kcal/mol)	Hydrogen Bonds	Hydrophobic Interactions	π-π Interactions	π-Cation Interactions	Salt Bridges
6B1E	CMNPD17868	−13.45	−73.08	Tyr547, Arg669, Glu205, Glu206	Tyr547, Phe357, Val207, Tyr666, Tyr662, Trp659, Val656, Val711, Tyr631, Trp629	Phe357	Tyr666	Arg125, Glu205, Glu206
	CMNPD13046	−13.35	−74.92	Arg125, Glu206, Val207, Ser630, Asp739	Trp124, Val207, Phe357, Tyr547, Trp629, Tyr631, Val656, Trp659, Tyr662, Tyr666, Val711, Ala743	Tyr666, His740		Glu205, Glu206
	Vildagliptin	−6.11	−53.57	Glu205, Glu206, Asn710	Phe357, Tyr547, Tyr631, Val656, Trp659, Tyr662, Tyr666, Val711			
5I7U	CMNPD17868	−11.68	−73.82	Glu206, Tyr662, Arg669	Val207, Phe357, Val546, Tyr547, Trp629, Tyr631, Val656, Trp659, Tyr662, Tyr666, Val711		Tyr666	Glu205, Glu206
	CMNPD13046	−12.79	−70.77	Arg125, His126, Glu205, Glu206, Tyr662, Asp739	Tyr547, Tyr631, Val656, Trp659, Tyr662, Tyr666, Val711, Ala743	Tyr666		Glu205, Glu206
	Vildagliptin	−7.05	−56.35	Glu205, Glu206	Phe357, Tyr547, Tyr631, Val656, Tyr662, Tyr666, Val711			
5T4E	CMNPD17868	−11.54	−82.49	Glu206, Ser209, Lys554	Val546, Tyr547, Trp629, Tyr631, Val656, Trp659, Tyr662, Tyr666, Val711		Arg125, Tyr666	Glu205, Glu206, Lys554
	CMNPD13046	−11.4	−79.27	Lys122, Glu205, Glu206, Asp545, Tyr631	Trp124, Phe357, Val546, Tyr547, Trp627, Trp629, Tyr631, Tyr666, Ala743, Tyr752		Lys554	
	Vildagliptin	−6.37	−50.96	Glu205, Glu206	Val207, Phe357, Tyr547, Tyr631, Trp659, Tyr662, Tyr666			

## Data Availability

The data presented in this study are available in the article and the supplementary materials.

## Supplementary Materials

The following are available online at https://www.mdpi.com/article/10.3390/md20120777/s1, Figure S1: Ligand interaction diagram of DPP-4 residues that interact with the docked ligand; Figure S2: Superimposition of the docked ligands in the active site of DPP-4.
