# Supplementary material for: A Molecular Modeling Investigation of the Therapeutic Potential of Marine Compounds as DPP-4 Inhibitors
Source: Mar Drugs. 2022 Dec 13;20(12):777. doi: 10.3390/md20120777 (PMC9788368; doi:10.3390/md20120777)
Supplement: Supplementary file 1 [file marinedrugs-20-00777-s001.zip › marinedrugs-2011468-supplementary.pdf]

A molecular modeling investigation of the therapeutic potential of marine compounds as DPP-4 inhibitors

Priya Antony[1,†], Bincy Baby[1,†], Hamda Mohammed Aleissaee[2] and Ranjit Vijayan[1,3,4 *]

[1] Department of Biology, College of Science, United Arab Emirates University, Al Ain, United Arab Emirates
[2] Department of Chemistry, College of Science, United Arab Emirates University, Al Ain, United Arab Emirates
[3] The Big Data Analytics Center, United Arab Emirates University, PO Box 15551, Al Ain, United Arab Emirates.
[4] Zayed Center for Health Sciences, United Arab Emirates University, PO Box 17666, Al Ain, United Arab Emirates.
[†] These authors contributed equally to this work.
* Correspondence to ranjit.v@uaeu.ac.ae (R.V.)

SUPPLEMENTARY MATERIALS

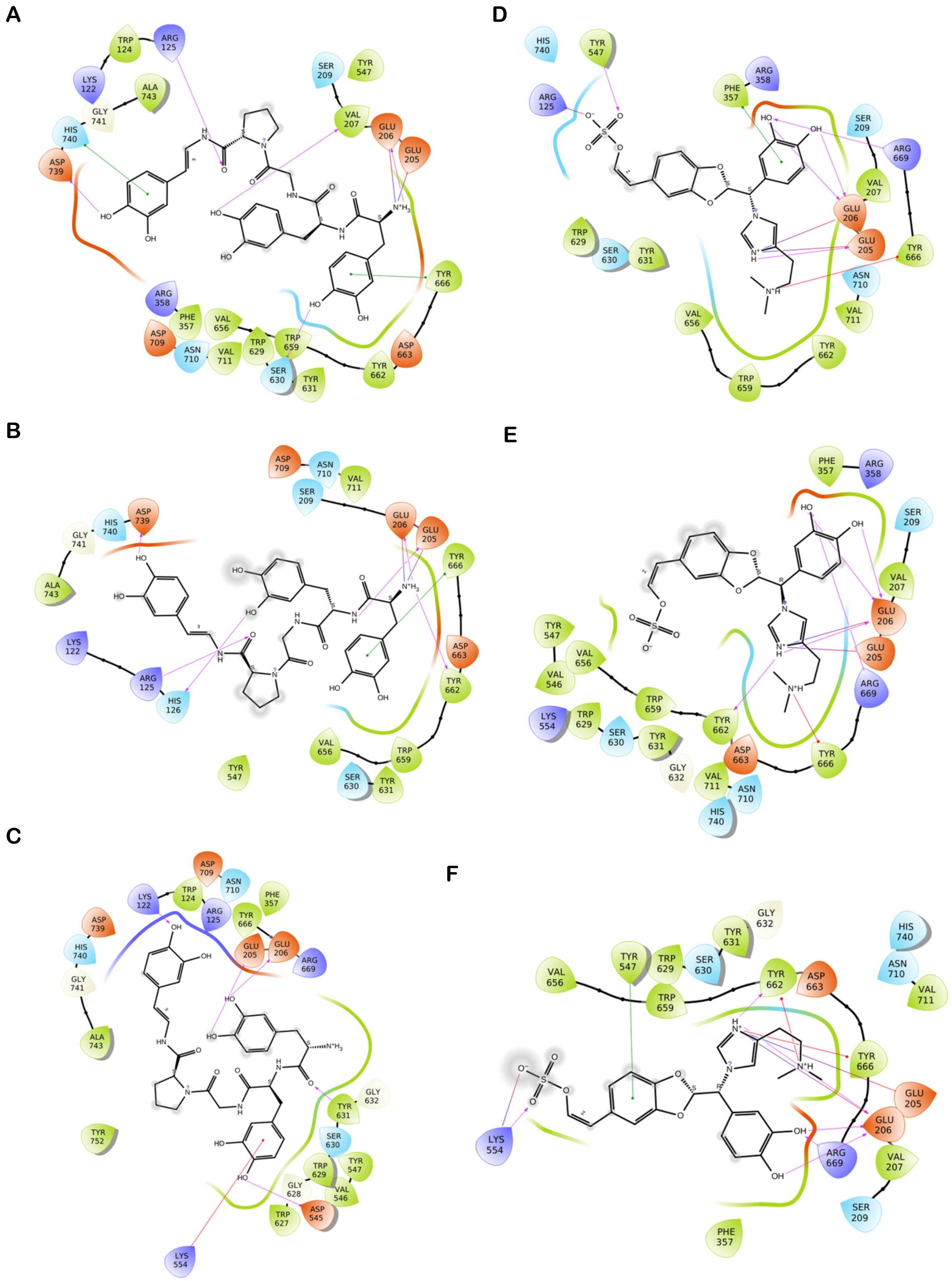

Figure S1. Ligand interaction diagram of DPP-4 residues that interact with the docked ligand in complexes: **(A)** 5I7U-CMNPD13046; **(B)** 5T4E-CMNPD13046; **(C)** 6B1E-CMNPD13046; **(D)** 5I7U-CMNPD17868; **(E)** 5T4E-CMNPD17868; **(F)** 6B1E_CMNPD17868 .

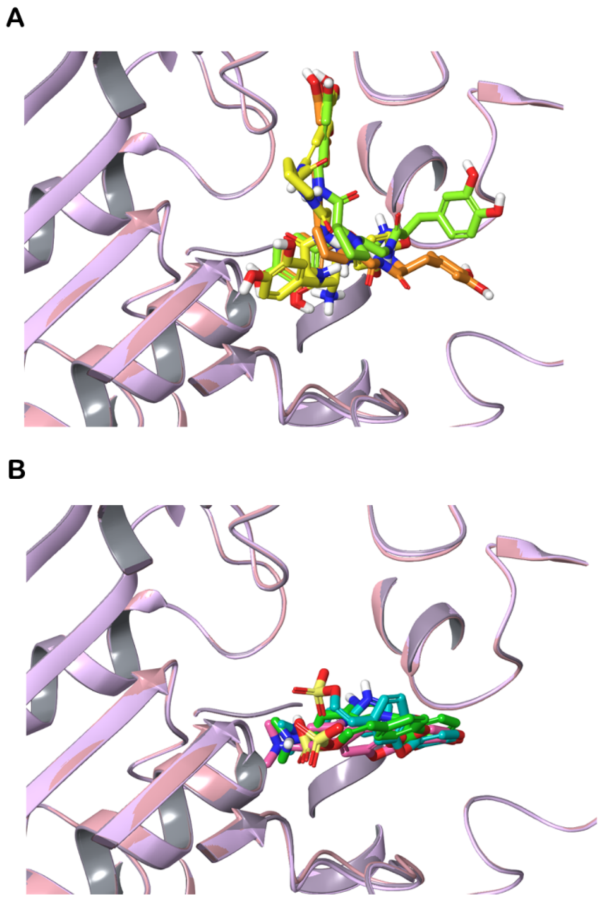

Figure S2. Superimposition of the docked ligands in the active site of DPP-4. **(A)** 6B1E-CMNPD13046 (orange), 5I7U-CMNPD13046 (green), and 5T4E-CMNPD13046 (yellow); **(B)** 6B1E-CMNPD17868 (cyan), 5I7U-CMNPD17868 (green), 5T4E-CMNPD17868 (pink).
